# A Fragile Balance: Does Neutrophil Extracellular Trap Formation Drive Pulmonary Disease Progression?

**DOI:** 10.3390/cells10081932

**Published:** 2021-07-29

**Authors:** Helena Block, Alexander Zarbock

**Affiliations:** Department of Anesthesiology, Intensive Care and Pain Medicine, University Hospital Muenster, 48149 Muester, Germany; helena.block@uni-muenster.de

**Keywords:** neutrophil extracellular traps, pneumonia, inflammation, COVID-19, influenza, community-acquired pneumonia, cystic fibrosis, chronic obstructive pulmonary disease

## Abstract

Neutrophils act as the first line of defense during infection and inflammation. Once activated, they are able to fulfil numerous tasks to fight inflammatory insults while keeping a balanced immune response. Besides well-known functions, such as phagocytosis and degranulation, neutrophils are also able to release “neutrophil extracellular traps” (NETs). In response to most stimuli, the neutrophils release decondensed chromatin in a NADPH oxidase-dependent manner decorated with histones and granule proteins, such as neutrophil elastase, myeloperoxidase, and cathelicidins. Although primarily supposed to prevent microbial dissemination and fight infections, there is increasing evidence that an overwhelming NET response correlates with poor outcome in many diseases. Lung-related diseases especially, such as bacterial pneumonia, cystic fibrosis, chronic obstructive pulmonary disease, aspergillosis, influenza, and COVID-19, are often affected by massive NET formation. Highly vascularized areas as in the lung are susceptible to immunothrombotic events promoted by chromatin fibers. Keeping this fragile equilibrium seems to be the key for an appropriate immune response. Therapies targeting dysregulated NET formation might positively influence many disease progressions. This review highlights recent findings on the pathophysiological influence of NET formation in different bacterial, viral, and non-infectious lung diseases and summarizes medical treatment strategies.

## 1. Introduction

The lung comprises different mucosal and alveolar compartments harboring resident immune cells maintaining a well-balanced milieu of protection versus potentially infectious inhaled pathogens. Lung infections may aggravate and turn into life-threatening diseases. Excessive neutrophil recruitment is a major risk factor, and the well-balanced activation of neutrophils is a prerequisite for an adequate immune response. Once pathogens have infiltrated the lung, epithelial cells, lung resident macrophages and dendritic cells (DCs) produce inflammatory mediators leading to immune cell recruitment, which occurs in a tightly regulated cascade. Activated neutrophils can fulfil numerous tasks to fight infection, such as degranulation, the release of reactive oxygen species (ROS), phagocytosis and the release of neutrophil extracellular traps (NETs). NETs are composed of extracellular decondensed chromatin in the majority of the nuclear but also with mitochondrial origin. The chromatin fibers are decorated with a variety of proteins, e.g., neutrophil elastase (NE), myeloperoxidase (MPO), histones, calprotectin, α-defensins, cathelicidins, and cytoskeletal proteins [1,2]. Initially, 24 proteins were identified following a PMA stimulation of neutrophils, but some studies extended this list to up to 50 different proteins and suggest a stimulus-dependent protein composition [3,4].

**Figure 1:**
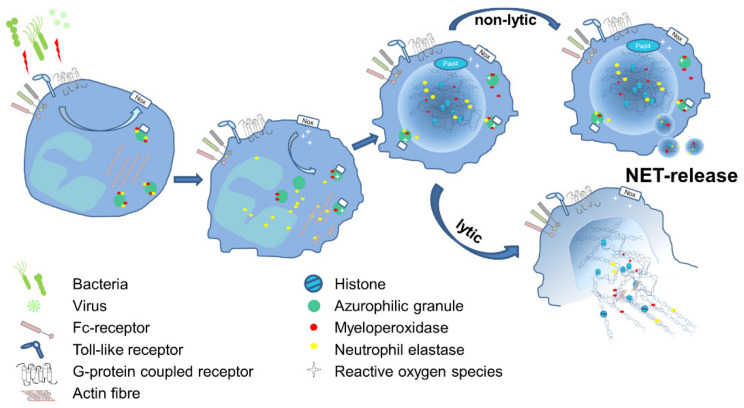
Schematic illustration of the essential steps of NET formation. Several pathogens are capable of inducing NET formation directly or via release of peptides or damage associated molecular patterns (DAMPs). Receptors like TLR, FcR or GPCR transmit signals into the cell and activate predominantly the NADPH-oxidase complex (NOX), which subsequently catalyzes production of reactive oxygen species (ROS). In azurophilic granules, NE gets released from the membranes in a ROS-dependent manner and translocates into the nucleus and in parallel degrades actin. NE activity results in the decondensation of chromatin, which is further supported by the PAD4-dependent citrullination of histones. The chromatin, decorated with microbicidal molecules like histones, MPO and NE, is released in the environment. This occurs either in a non-lytic procedure, where DNA fibers are suggested to be released via vesicles, or in a lytic process, followed by the breakdown of the nuclear envelope and the cell membrane, ending with cell death.

The underlying mechanisms leading to NET formation show variable characteristics, and studies demonstrated that the signaling pathways vary depending on the respective stimulus. NET formation can be stimulated via G protein-coupled receptors (GPCRs), chemokine and cytokine receptors, Toll-like receptors (TLR), and Fc receptors (FcR). The subsequent downstream signaling comprises mostly the activation of the NADPH oxidase (NOX) complex, but exceptions were also described [5]. Upstream of oxidant production, the molecules Raf-MEK-ERK have been shown to be involved [6]. Cytoskeletal rearrangement [7] and glycolytic ATP production [8] are both required for NET formation and are dependent on ROS produced in the context of mitochondrial dysfunction and NOX activation [9]. ROS initiates the dissociation of NE from a membrane-associated complex into the cytosol and activates its proteolytic activity in an MPO-dependent manner. Subsequently, NE degrades F-actin to arrest actin dynamics followed by translocation into the nucleus, where NE and MPO drive chromatin decondensation and histone cleavage [10,11], which can be supported by PAD4-dependent histone citrullination [12] (Figure 1). Nevertheless, NE- and PAD4-independent pathways have been described, too [13,14]. Van Avondt and colleagues demonstrated that the inhibition of the signal inhibitory receptor on leukocytes-1 (SIRL-1) could prevent NET production without affecting oxidant production [15]. Cell cycle proteins [16] support nuclear envelope breakdown followed by the release of chromatin into the cytosol, where nuclear and cytosolic proteins are mixed [17]. The final cell lysis and NET release involves Gasdermin D (GSDMD), which is able to form pores in granule and plasma membranes [18,19]. This kind of NET formation ends up with cell death and is often described as lytic NET release or NETosis and occurs within a rather long time frame of three to eight hours. In contrast, the non-lytic NET release can be observed rapidly after 5–60 min of stimulation. Here, neutrophils do not undergo cell death, which was observed for neutrophils in close contact with activated platelets [20,21] or in response to *Staphylococcus aureus* infections [22]. Similar to NETosis, the non-lytic NET formation also involves the translocation of NE to the nucleus, histone citrullination, and chromatin decondensation [23]. Conversely, the membrane does not disintegrate, and the protein-decorated chromatin is released via vesicles [24] (Figure 1). Even the remnants of non-lytic NET formation, cytoplasts, are able to keep their mobility and fulfill important functions, such as phagocytosis, the activation of DCs, and the release of cytotoxic molecules [24,25,26].

The direct capture and clearance of pathogens by neutrophils can occur via phagocytosis or NET formation. Branzk and colleagues published a study suggesting that the pathogen size determines the neutrophil response. Large pathogens such as hyphae initiate NET formation, whereas small single-cell bacteria are eliminated via phagocytosis. Both events do not occur at the same time, since phagocytosis can prevent NET release by inhibiting NE translocation to the nucleus [27]. It is reasonable to extend this finding to cocci- or biofilm-forming bacteria, such as *S. aureus*, which also induce NET formation rather than phagocytosis.

**Figure 2:**
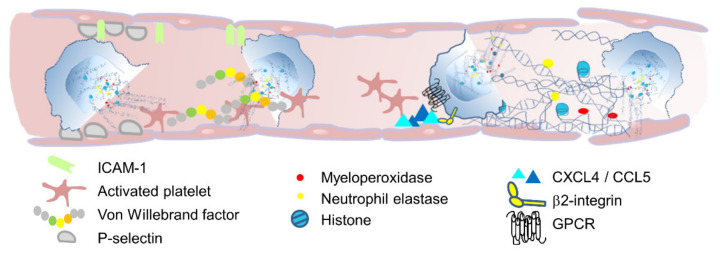
Schematic illustration of how NETs might contribute to vaso-occlusion and endothelial damage. NETs are able to promote vaso-occlusion, initiated by von Willebrand production in platelets, which induces the upregulation of intercellular adhesion molecule-1 (ICAM-1), further strengthening neutrophil–endothelium interactions. Platelet-dependent NET formation requires the heterodimerized CXCL4 and CCL5, as well as the simultaneous stimulation of GPCRs and integrins. NET-derived NE cleaves the coagulation-inhibiting tissue factor pathway inhibitor (TFPI) and, in parallel, activates platelet receptors to increase platelet accumulation. NET-decorated proteins further contribute to tissue damage. Histones especially exhibit cytotoxic effects, disturbing the endothelial integrity.

Taken together, exteriorized chromatin fibers are able to entrap and alleviate the elimination of bacteria, fungi, protozoa, and even viruses [1,2,28,29,30,31]. Nevertheless, these properties are not only deleterious for invading pathogens but can also harm the host as well. NETs are able to kill epithelial and endothelial cells [32], and histones especially have a cytotoxic capability disturbing membrane integrity [33,34]. Additionally, NETs are able to promote vaso-occlusion, initiated by the hypoxia-induced release of von Willebrand factor (VWF) and endothelial P-selectin, resulting in neutrophil recruitment and activation [35,36]. Another proposed mechanism is the P-selectin-dependent neutrophil and platelet recruitment. Here, neutrophils initiate thromboxane A2 production in platelets, which induces the upregulation of intercellular adhesion molecule-1 (ICAM-1), further strengthening neutrophil–endothelium interactions [37]. This process induces NET formation implicating platelet-derived high mobility group protein B1 (HMGB1), ROS, and integrins [36,38]. Platelet-dependent NET formation requires the pro-inflammatory heterodimerized CXCL4 and CCL5, as well as the simultaneous stimulation of GPCRs and integrins [39]. NETs can further contribute to vessel occlusion through recruitment of factor XIIa, which mobilizes Weibel-Palade bodies containing VWF, P-Selectin, and factor XIIa [38,40]. Extracellular NET histones bind VWF and fibrin to recruit red blood cells and platelets [41,42], whereas NE cleaves the coagulation-inhibiting tissue factor pathway inhibitor (TFPI) and, in parallel, activates platelet receptors to increase platelet accumulation [43,44]. Thrombotic events can disturb the microcirculation particularly in the lungs, resulting in small pulmonary vessel occlusion [42,45], and, therefore, contribute to the pathogenesis of numerous diseases [46] (Figure 2). Although accumulated NETs especially seem to worsen disease outcome, the mechanisms of NET resolution and how NETs influence the resolution of inflammation are poorly understood.

Beside the degradation of NETs through DNases, some studies also suggest a contribution of macrophages to NET resolution and degradation. In vitro experiments with human monocyte-derived macrophages and PMA-stimulated human neutrophils demonstrated that macrophages are able to internalize NETs in a cathelicidin LL37-dependent manner and degrade DNA via TREX1/DNAseIII. In this setting, DCs contribute to extracellular NET degradation by DNase1L3. Here, cytokine profiling indicated that NETs alone are non-inflammatory but could also be immune modulatory in presence of LPS [47]. In contrast, Apel and colleagues describe a mechanism where phagocytosed NETs are able to activate the innate immune sensor cyclic GMP-AMP synthase (cGAS), thereby inducing the production of pro-inflammatory type I interferons [48]. Another study suggested a dual, phenotype-dependent role of macrophages: in the early phase, M2 macrophages induced a pro-inflammatory response and sustained the inflammatory state. In the second phase, M1 macrophages underwent cell death with nuclear decondensation, which took place in a PAD4-dependent manner and resulted in a local release of extracellular DNA. In the late phase, M1 macrophages degraded this DNA in a caspase-activated DNase-dependent manner resulting in the clearance of extracellular DNA within 24 h [49]. The release of nuclear DNA by macrophages or monocytes has been described by different groups and is referred to as macrophage extracellular traps (METs). They are also attributed to offer anti-microbial functions and contribute to pathology. Nevertheless, the respective studies show conflicting results, which has been discussed in more detail before [50].

NET aggregates (aggNETs), first described in a murine gout model [51], are formed at sites of high neutrophil density and contain enzymes that cleave, bind or modify autologous and foreign macromolecules. AggNETs are able to sequester and degrade histones and, thus, attenuate their cytotoxic effect on epithelial cells [52]. This process was executed by at least two aggNET-borne serine proteases, NE and PR3. Further, they are capable of resolving inflammation by the proteolytical degradation of inflammatory cytokines and chemokines [51,53]. Nevertheless, the physiological relevance of these proposed mechanisms remains elusive, and further work is required to shed light on the mechanisms of NET resolution and degradation.

This review focuses on the role of NET formation during virus-induced lung infections, as well as primary and secondary bacterial infections, and summarizes possible therapeutic interventions.

## 2. The Role of NETs in Virus-Induced Lung Diseases

Up to the beginning of 2020, NET formation in virus-induced lung diseases was of minor interest compared to bacterial lung infections. The current COVID-19 pandemic spotlighted NET formation within this disease, since several studies demonstrated a strong contribution of NETs to thrombosis and pulmonary vessel occlusion in COVID-19 patients. Here, we summarize recent research data and put them in context with other virus-induced diseases, such as respiratory syncytial virus (RSV) and influenza, with a special focus on NETs in the lung.

### 2.1. COVID-19

Similar to other SARS viruses, SARS-CoV-2 enters cells through angiotensin-converting enzyme 2 receptor (ACE2), expressed on renal and pulmonary endothelial cells [54,55]. Infected cells release paracrine factors influencing epithelial cells, neutrophils, and pneumocytes [56] and recruit further immune cells. The damaged lung during COVID-19 displays deformed capillaries, alveolar capillary damage, fluid-filled alveoli, hemorrhage, fibrin deposition, signs of compensatory neovascularization, and immune cell infiltration [57,58,59,60], which are altogether responsible for respiratory symptoms and shortness of breath. Disease severity was correlated to neutrophilia, indicating a direct contribution [61]. NET components, such as MPO-DNA, and citrullinated histone H3 are increased in patients with lung distress and higher severity of disease [62] and might serve as prognostic factors. In fact, viable SARS-CoV-2 can induce NET formation in human neutrophils in a dose-dependent manner [63] and requires virus replication, serine protease activity, and ACE2. NE, which is associated with NETs, is supposed to increase the susceptibility to SARS-CoV-2 infection by facilitating cell entry and virulence [64].

Histopathological lung analysis of SARS-CoV-2-related acute respiratory distress syndrome (ARDS) patients revealed small pulmonary vessel occlusion by NET aggregates, which was less often observed in histological analysis of influenza-induced pneumonia [45,65]. COVID-19-associated NETs are decorated with TF, which has been attributed to complement activation [66]. Complement activation may trigger coagulopathy and cytokine storm, both described as critical symptoms during COVID-19 [67]. Complement deficiencies in COVID-19 patients seem to be protective [67], whereas macular degeneration, which is associated with complement activation or a history of coagulation disorders, are rather poor prognostic factors [68].

Additionally, neutrophil activation markers as well as neutrophil–platelet aggregates are strongly elevated in patients with severe disease, whereas cases of intermediate severity displayed a hypo-reactive neutrophil phenotype and exhausted platelets [69]. The severe course has been linked to dysregulated immunothrombosis, resulting in ARDS and systemic hypercoagulability.

Formed NETs can further activate and damage endothelial cells, weakening the endothelial barrier integrity [70,71,72]. Circulating NET components can therefore reach other organs and, once accumulated, trigger microvascular thrombosis [45,66,73]. NET components with a high density of cationic residues, such as histone H4, can bind to negatively charged plasma membranes, which result in cell lysis through pore formation, finally leading to inflammation and tissue damage [74].

### 2.2. Respiratory Syncitial Virus

RSV is one of the most common causes for bronchiolitis in young children worldwide. At the age of 3, nearly all children have acquired at least one infection with the virus [75,76], and estimations suggest it is responsible for more than 3 million hospitalizations and almost 200,000 deaths per year for children <5 years. Characteristic symptoms are massive neutrophil accumulation in the lungs and occlusion of small airways by DNA-rich mucus plugs, resulting in coughing, wheezing, and labored breathing [77]. RSV activates neutrophils, induces IL-8 secretion, degranulation [78], and inhibits neutrophil apoptosis in a phosphoinositide 3-kinase (PI3K) and nuclear factor κb (NF-κB)-dependent manner [79].

The DNA content fosters the viscosity of the mucus, and it derives from necrotic inflammatory and epithelial cells, but also from RSV-induced NETosis, as demonstrated independently from two different groups [80,81]. Muraro and colleagues revealed that RSV induces lytic NET formation with 3 h of incubation dose dependently in human neutrophils in vitro. They suggest a mechanism where RSV fusion protein induces NET formation via TLR4 activation, further signaling via PI3K/AKT, ERK, p38-MAPK, NADPH-oxidase, and PAD4 [81,82]. Further evidence that NET components in the mucus contribute to worsening inflammation was provided by demonstrating that the bronchoalveolar lavage fluid (BALF) of severe RSV-induced lower respiratory tract disease (LRTD) in children contains NETs, involving NE, and citrullinated histone H3 [80]. In vitro assays revealed that NET formation and RSV trapping resulted in a reduced infection of epithelial cells. Nevertheless, only a minor fraction of NET-containing small airway occlusions in lung tissue sections of bRSV-LRTD in calves contained RSV antigens, suggesting further NET-inducing stimuli. This further raises the question of which events can turn the formerly positive NET function, the trapping of virus and diminishing of further infection, into something detrimental. The detailed investigation of the underlying mechanisms is a prerequisite to identify potential targets for effective therapies.

### 2.3. Influenza

Influenza A is a recurring infection with varying severity. Clinical symptoms include fever and upper respiratory tract complications, such as runny nose, cough, and sore throat [83]. Patients at high risk for complications are of older age or have pre-existing medical conditions. Complications include pneumonia, bronchiolitis, toxic shock syndrome, seizures, and bacterial pneumonia. Infection occurs via the carbohydrate-binding protein haemagglutinin (HA) and the enzyme neuraminidase (NA) to cleave the glycosidic bonds of the sialic acid residues of plasma membranes [84].

Similar to RSV, excessive neutrophil infiltration of the lung is a characteristic feature of influenza A [85,86,87]. Influenza A infection of mice revealed that neutrophil recruitment is promoted by the epithelial and endothelial expression of G-CSF and CXCL4 [88,89], and it depends on CXCR2 [90]. Beside the positive disease-limiting effects of neutrophils [86,87,91] and the ability to predispose neutrophils for possible secondary bacterial infections [92], the abundance of neutrophils can also exert harmful functions. Transcriptional analysis revealed a chemokine-driven feedforward circuit, potentially leading to lethal influenza infection [93]. Additionally, it was demonstrated that influenza infection leads to C3 release of platelets, triggering NET formation in a TLR7-dependent manner [94]. In influenza H1N1-infected mice, lung-recruited neutrophils showed an upregulation of several chemokine receptors (CCR1, CCR2, CCR3, CCR5, CXCR1, CXCR3, and CXCR4) compared to circulating neutrophils. In vitro experiments suggest their possible role in modulating chemotaxis, phagocytosis, and NET formation [95]. Recent data suggest subtype-specific differences, e.g., that subtype H1N1 induces NET formation with rather less chemokine and cytokine transcription, whereas H5N1 does not trigger NET formation but induces a higher expression of inflammatory cytokines [96].

Excessive NET release was correlated with poor outcome following influenza A infection [97]. Similar to RSV or COVID-19, NETs decorated with histones and MMP-9 were found in mice infected with influenza A-H1N1 inducing alveolar capillary damage and an obstruction of small airways [98], but they were also attributed to protective features in the liver following poxvirus infection [31]. Nevertheless, in mice deficient in PAD4, influenza A infection severity is not altered compared to WT mice. In contrast, in vitro studies suggest an anti-viral role of α-defensin, a cationic antimicrobial molecule. Here, cell treatment with human α-defensin-1 results in a significant inhibition of influenza virus replication and viral protein synthesis, probably through a protein kinase C-dependent mechanism [99]. Taken together, the well-balanced formation of NETs is essential for a mild course of disease and for an early defense against secondary bacterial infections. Inappropriate NET formation seems to be one of the main factors for a poor prognosis during influenza infection.

## 3. The Role of NETs in Bacteria-Associated Lung Diseases

Community-acquired pneumonia (CAP) is one of the most common infectious diseases and remains a burden worldwide. It is responsible for hospitalization and represents a cause of considerable morbidity and mortality. The development of CAP and secondary bacterial infections of the lung occurs likely by translocation or aspiration of nasal colonizing bacteria. These bacteria usually act as commensals but can infect the lung upon the expression of a wide array of species-specific virulence factors. Preceding viral infections can potentiate lung infection by damage and alterations in pulmonary antibacterial immunity. The most frequent causes of CAP admitted to intensive care units are infections with *Streptococcus pneumoniae* and *Staphylococcus aureus* [100]. In this section, we focus on the role of NETs during these bacterial infections. The infection with *Pseudomonas aeruginosa* is also of interest and is discussed later in the context of cystic fibrosis.

### 3.1. Streptococcus Pneumoniae

*S. pneumoniae*, also known as pneumococcus, is a Gram-positive diplococcus and is one of the main causes for bacterial pneumonia. It colonizes the mucosa of the human nasopharynx. Aspiration of nasopharyngeal secretions enables the invasion of the lung parenchyma, leading to pulmonary infection [101]. Following the secretion of inflammatory chemokines, neutrophils are recruited and fight infections by phagocytosis with subsequent degradation by proteases such as NE and cathepsin G stored in azurophilic granules [102] and by forming NETs with significant antibacterial activity against *S. pneumoniae* [103]. Invasive serotypes often express polysaccharide capsules, which confer resistance against phagocytic killing [104] and also reduce entrapment within NETs. NET formation was directly correlated with the thickness of the pneumococcal capsule, further augmenting disease severity in mice [105]. However, a higher incidence of NET components in CAP patients was associated with increased mortality [106]. Virulent pneumococci release EndA, a membrane-localized endonuclease capable of degrading NETs in vitro, contributing to host response evasion [107,108]. Additionally, EndA can foster bacteria spreading from the upper airways to the lungs and further to the bloodstream of infected mice [107]. Furthermore, the extracellular vesicle-associated endodeoxyribonuclease TatD is also capable of degrading NETs, and TatD-deficient pneumococci displayed compromised virulence with improved lung pathology during murine sepsis compared to the wild-type strain [109].

However, the release of NE by neutrophils either through the pneumolysin-induced leakage of neutrophils or the degradation of NETs is not only detrimental to invading microorganisms but also to the host. It degrades extracellular matrix components, such as elastin, fibronectin, collagen, and proteoglycan [110,111]. Additionally, it reduces the phagocytic activity of macrophages [112], impairs the pulmonary endothelial barrier, and injures alveolar epithelial cells [113], altogether contributing to host tissue damage [114]. NE is further capable of influencing the immune response by cleaving cell surface receptors, such as TLR2, TLR4, CD14, TNFR, and C5a [115,116,117], and degrading multiple inflammatory cytokines and chemokines, such as interleukin (IL)-1β, IL-2, IL-6, IL-8, IL-12p40, IL-12p70, and TNFα [115,117,118]. Recent studies demonstrate that NE cleaves human leukocyte antigen class II molecules in both cultured macrophages and in vivo mouse models, indicating that NE may disrupt antigen presentation and T-cell activation [119]. In contrast, NE cleaves and activates MMP-9, which may also have a destructive role in lung diseases [120]. Collectively, these data imply that NE may assist the dissemination of pneumococci by cleaving a variety of host immune proteins and inducing lung injury. Whether the inactivation of inflammatory cytokines also has a beneficial effect and prevents overwhelming leukocyte recruitment remains to be determined. Additionally, *S. pneumoniae* protects itself from NET killing by the incorporation of D-alanine into surface lipoteichoic acids (LTAs), which results in a positive charge of the bacterial membrane, blocking cationic peptides as histones [121]. In mice, lung infection with equal amounts of WT pneumococci and pneumococci lacking the dlt operon, encoding the respective enzymes for D-alanylation of LTA, displayed an increased dissemination of WT pneumococci in lungs and blood, indicating that D-alanylation supports NET evasion [121]. In summary, pneumococci successfully developed several strategies to evade clearance by neutrophils. As an unfortunate circumstance, capsules as part of this strategy also trigger additional NET formation, which additionally contributes to disease severity, finally resulting in increased mortality.

### 3.2. Staphylococcus Aureus

*S. aureus* emerged to an important cause of CAP with severe complications requiring intensive care and resulting in high mortality [122]. The rise of methicillin-resistant *S. aureus* (MRSA) even increased the threat of this pathogen. Preceding influenza infections increased the risk for mortality [123].

Especially known for the production of toxins as well as the generation of biofilms, both contribute to the successful invasion of the host while evading the hosts immune response [124,125,126]. The virulence depends on several factors, influencing the transition from colonization to infection, adhesion, iron acquisition, immune evasion, expression of pore-forming toxins, and metabolic regulators, as extensively reviewed elsewhere [127].

*S. aureus* infection of the lung leads to the recruitment of neutrophils combating infection. *S. aureus* has evolved multiple mechanisms of inhibiting neutrophil phagocytosis by interfering with complement activation and preventing Fc receptor binding [128,129,130,131]. Even if *S. aureus* is phagocytosed, it can survive inside neutrophils. Therefore, a rapid intervention to combat *S. aureus* infection might be more effective. Interestingly, *S. aureus* is able to induce the rapid, non-lytic, NADPH-independent NET formation [24]. Following DNA extrusion, cytoplasts are still able to migrate and phagocytose in vitro. Pilsczek and colleagues [22] revealed that Panton–Valentine leukocidin (PVL) is a potent NET inducer secreted by *S. aureus*. PVL is further able to lyse neutrophils, which can in turn neutralize PVL by α-defensins, which are part of NETs [132].

*S. aureus* expresses nucleases that are able to degrade NETs, conferring resistance to NET-mediated killing. In a murine mouse model, nuc-deficient *S. aureus* were significantly more susceptible to extracellular killing by neutrophils, whereas nuclease expression resulted in delayed bacterial clearance and increased mortality [133]. Additionally, in vitro assays indicated that the NET degradation product 2‘-deoxyadenosine (dAdo) is able to induce apoptosis in macrophages, further corroborating immune cell evasion of *S. aureus* [134]. The balance between the appropriate defense against pathogens and the destruction of lung barrier function is fragile, which was also demonstrated in murine pneumonia experiments investigating the impact of different levels of NETs during lung infection with *S. aureus* [135]. A decreased amount of NETs reduced lung injury and improved survival after DNase I treatment or with partial protein arginine deiminase 4 deficiency (*PAD4^+/–^*). Complete PAD4 deficiency (*PAD4^–/–^*) reduced NETs and lung injury but was counterbalanced by an increased bacterial load and inflammation. In line with this, mice deficient in the lipoxin receptor (*Fpr2^–/–^*) produce excessive NETs resulting in increased lung injury and mortality. In this context, samples from critically ill patients with increased plasma NETs were associated with ARDS severity and mortality, and lower plasma DNase I levels were associated with the development of sepsis-induced ARDS [135].

Several studies indicate a role of both PVL as well as nucleases during biofilm formation. Biofilm formation is a very effective measure to evade several immune defense mechanisms and enables long persistence of bacteria resulting in chronic disease.

During the early stages of biofilm formation, *S. aureus* produces immune modulators, such as staphylococcal complement inhibitor (SCIN), chemotaxis inhibitory protein of staphylococci (CHIPS), and formyl peptide receptor-like 1 inhibitor (FLIPr), as well as early production of thermonuclease [136,137]. These immunomodulators facilitate the defense of developing biofilms against the host early immune responses. To further corroborate these mechanisms, *S. aureus* biofilms have been shown to foster NET formation at the expense of other neutrophil defense mechanisms by releasing PVL and γ-hemolysin AB [138]. Competing with nuclease secretion by *S. aureus*, NETs are not able to efficiently combat infection. Additionally, long persisting *S. aureus* has been shown to be able to adapt to neutrophil-rich environments by increasing nuclease expression to evade NET killing, as it was demonstrated in airway isolates of cystic fibrosis patients [139].

The plethora of evasion mechanisms of *S. aureus* to the immune response underlines the importance of a fast and efficient treatment in the early phase of disease. Once *S. aureus* has manifested, the positive function of NETs declines and their deleterious role worsens disease outcome.

### 3.3. Cystic Fibrosis and Pseudomonas Aeruginosa

Cystic fibrosis (CF) is a genetic disease caused by mutations of the cystic fibrosis transmembrane regulator (CFTR) gene, a protein member of the ATP-binding cassette (ABC) transporter superfamily. It functions as a chloride channel that controls the transport of ions and water across epithelial tissues. The clinical manifestations of the CF are predominantly chronic airway infection and inflammation, which lead to a progressive decrement in lung function, pancreatic insufficiency, malnutrition, and hepatobiliary symptoms [140]. *Pseudomonas aeruginosa* is the major pathogen involved, causing thick mucus and reduced mucociliary clearance favoring chronic bacterial infections, including neutrophil-rich airway inflammation followed by increased rates of morbidity and mortality [141,142]. Further, *P. aeruginosa* induces neutrophil recruitment. Once migrated to the site of infection, neutrophils perform their classical anti-bacterial functions, including phagocytosis, ROS production, degranulation, and NET formation [143,144]. However, in CF patients, neutrophils fail to eliminate *P. aeruginosa* invasion but rather contribute to tissue damage [145,146], which is further supported by decreased apoptosis [147,148]. NETs have been shown to be present in the sputum, contributing to an increased mucus viscosity [149,150] and, additionally, to tissue damage with decreased pulmonary function [143,144]. When cell-free DNA is enriched in the sputum, patients display diminished lung function compared to patients showing mild symptoms, indicating that the airway obstruction is a result of accumulated NETs [3,151,152,153]. Moreover, it has been reported that NET components, such as MPO, NE and histones, can damage epithelial, endothelial, and connective tissues, worsening the lung pathology [32,149,154].

An impaired clearance of NETs from the airways might also contribute to pathogenesis and depends on several factors, including mucociliary clearance, DNase activity, and phagocytosis by macrophages. The phagocytosis is facilitated by pre-degradation by DNase and does not result in pro-inflammatory cytokine secretion [47,155]. CFTR-deficient macrophages have an abnormally high intraphagolysosomal pH, which was shown to alter bactericidal activity and might also impair NET resolution [156].

*P. aeruginosa* developed strategies to evade neutrophils and their respective immune responses. Upon infection of the patients’ lung, this pathogen can migrate to areas with low oxygen concentrations where only few immune cells can exert their defense function [157]. Additionally, it has been shown that biofilm formation promotes excessive production of alginate that allows the escape of bacteria to neutrophil degranulation and phagocytosis [158].

In order to attenuate NET production and resist NET-mediated killing, *P. aeruginosa* can express surface sialic acids that are capable of binding and inducing signaling through neutrophil Siglec-9, further suppressing the oxidative burst and, thus, NET formation [159]. The authors in this study showed that treatment with sialidase or the use of *P. aeruginosa* strains lacking sialic acids led to increased NET production compared with sialic acid-positive strains. Furthermore, it was demonstrated that paired *P. aeruginosa* isolates from patients with CF at early and late stages of disease developed resistance to NET-mediated killing over time, which corresponded to the development of the mucoid, alginate-rich phenotype [157]. Nevertheless, the same study revealed that alginate overexpression did not increase survival upon incubation with PMA-treated neutrophils. This might be due to additional yet unknown defense mechanisms conferring NET evasion. Alternatively, this might also indicate that PMA-induced NETs may differ in their capability to combat pathogens compared to NETs that were formed in response to physiological stimuli.

Additionally, *P. aeruginosa* can overexpress genes controlled by the two component systems PhoPQ and PmrAB that sense Mg^2+^ limitation and, at the same time, encode mechanisms to effectively obtain the ion so that it cannot be complexed by NET structures [158,160,161]. Furthermore, bacteria are capable of regulating genes that allow them to tolerate the toxicity of extracellular DNA and their components [162].

Taken together, NETs during CF have only a limited anti-bacterial effect. Additionally, the prolonged survival of neutrophils in addition to the reduced resolution of NET structures essentially contribute to exacerbated inflammation. Further studies are required to elucidate the interplay between NETs and pathogens during CF.

### 3.4. Chronic Obstructive Pulmonary Disease

Chronic obstructive pulmonary disease (COPD) is one of the leading causes of death worldwide and a major cause of mortality in adults [163]. It is characterized by airflow limitation by narrowing of the small airways combined with emphysematous destruction of the alveoli. Chronic exposure to cigarette smoke contributes to COPD pathogenesis, where it can induce neutrophil retention within the airways [164]. In general, the degree of neutrophilia correlates with COPD severity [165,166], exacerbations [167], and disease progression [168]. Neutrophil recruitment into the sputum accounts for approximately one-third on CXCL8 [169], but also other CXCR2 ligands, such as CXCL1 and CXCL5, are elevated in COPD sputa, airway fluids, and bronchial tissues [166,170,171]. CXCR2 is upregulated in exacerbations of COPD where its expression co-localizes with the accumulation of airway mucosal neutrophils [170].

Several studies [172,173,174] observed NET formation in the sputum from both stable and exacerbated COPD patients using qPCR, ELISAs, and confocal fluorescent and electron microscopy, respectively. Elevated levels of sputum NETs were negatively associated with lung function and, additionally, COPD symptoms and PAD4 gene expression were found to be upregulated in neutrophilic compared to non-neutrophilic COPD patients [173]. Furthermore, increased NET formation in the airways of COPD patients was associated with disease severity [175]. This study suggested a relationship between sputum-enriched NETs and non-eosinophilic COPD exacerbations and reduced bacterial diversity accompanied by an abundance of *Haemophilus* species. Interestingly, the phagocytic capacity of neutrophils to engulf bacteria ex vivo was impaired in cells derived from patients with high sputum NET complexes or in the neutrophils of healthy donors incubated with the soluble sputum of COPD patients [175]. Since phagocytosis is more efficient in bacterial clearance, its suppression might also contribute to exaggerated inflammation and recurrent infections. This is further underlined by a study that demonstrated that COPD patients are highly susceptible to recurrent bacterial infection following infection with respiratory viruses, which are also known to induce NETs [176].

Taken together, these findings indicate a strong negative impairment of NETs on COPD and, therefore, also identify them as a promising therapeutic target. For a more detailed view on NETs in COPD, please see the recently published review by Trivedi and colleagues [177].

## 4. Pathogenic Fungal Lung Infection—Aspergillosis

Infection with pulmonary fungal pathogens is a severe clinical problem, especially in patients with compromised immune functions. Opportunistic fungi, including *Aspergillus* with invasive aspergillosis [178,179,180], *Cryptococcus* with cryptococcosis [181,182,183], *Pneumocystis* with pneumonia [184], and endemic fungi [185,186], are the main sources of fungal infections in the lungs of humans. *Aspergillus* mold is one of the most common fungal species, which is able to sporulate with released airborne conidia. With a size of 2–3 µm, they are small enough to infiltrate human airways and pulmonary alveoli, causing a spectrum of diseases [178,187]. Within the early phase of infection in healthy individuals, it is assumed that neutrophils restrict the tissue invasion of hyphae [188], whereas inhaled conidia are eliminated by alveolar macrophages [189,190,191]. Immunocompromised individuals exhibit tissue invasion by fungal hyphae due to the incomplete killing of inhaled fungal conidia [192]. Upon recognition of fungal pathogens, innate immune cells are activated by pathogen-associated molecular patterns (PAMPs) via specific pattern recognition receptors (PRRs) on the surface to trigger further intracellular signaling cascades. The PRRs involved in fungal detection identified to date include TLRs, C-type lectin receptors and NOD-like receptors [193,194].

As described before, neutrophils were suggested to sense microbe size and selectively release NETs in response to large pathogens such as *A. fumigatus* hyphae or large aggregated conidia but not in response to small single conidia [27], most probably to compensate for the inefficient phagocytosis of larger pathogens.

Indeed, investigating a murine model of pulmonary aspergillosis revealed that neutrophils are able to form NETs in response to *A. fumigatus*, in particular close to [195] developing clusters of fungi with outgrowing hyphae, whereas conidia are rather engulfed by neutrophils [195]. In vitro, NETs display fungistatic activity and are hypothesized to prevent fungal dissemination [29,195]. In response to Aspergillus, the inhibitory function of NETs has been shown to be mediated by calprotectin [29] and the release of long pentraxin 3, a pattern recognition receptor that activates complement and facilitates pathogen recognition [196]. The protective function of NETs was further underlined by investigating pulmonary aspergillosis in *p47phox^−/−^* mice, which failed to generate NETs and developed progressive pneumonia [197]. In contrast, another study induced invasive pulmonary aspergillosis in mice and demonstrated that *Pad4*^−/−^ mice revealed a lower fungal burden in the lungs, accompanied by a reduced acute lung injury, and less TNFα and citH3 compared to wild-type controls [198]. These findings suggest a detrimental role of NETs contributing to tissue damage and limiting the control of fungal outgrowth. However, the NET-mediated ability to combat fungal invasion remains controversial [2,199], but it should be noted that the exact mechanisms of fungal killing in mice can actually be different from those observed in humans [200].

Patients with neutropenia or hematologic malignancy, as well as those who suffer from chronic granulomatous disease (CGD), are predisposed to Aspergillus infection [201]. CGD patients have impaired phox function, resulting in poor NET production and reduced neutrophil activity [202]. In this context, *A. nidulans* emerges as a major pathogen, often resulting in refractory, disseminated disease [203]. In a clinical study involving a patient with CGD suffering from refractory invasive *A. nidulans* infection, Bianchi and colleagues suggested a link between the production of NETs and the resolution of invasive aspergillosis [204]. This idea was further underlined by in vitro experiments that demonstrated that phox-deficient neutrophils lack activity against *A. nidulans* conidia and hyphae. Genetically complementing phox function restored both NET production and antifungal activity. Furthermore, administration of gene therapy providing phox activity rapidly cured the patient with treatment-refractory *A. nidulans* infection [205].

NET evasion by *A. fumigatus* was linked to the expression of galactosaminogalactan (GAG), an α-1,4-linked linear heteroglycan composed of various combinations of galactose and N-acetyl-galactosamine [206,207,208]. Disruption of GAG attenuates both biofilm formation and virulence [208,209]. Its protective role against NETs was suggested to be mediated by its positive charge, which is able to bind to the cationic antimicrobial peptides or histones on NETs [210].

Taken together, the current knowledge about the role of NETs during fungi-induced lung diseases is rather scarce. Additionally, some contradictory studies concerning human and murine neutrophils exist. Nonetheless, investigating exactly these differences will probably generate valuable information about NET formation as a host defense mechanism combatting pathogenic fungi.

## 5. NET-Targeting Therapies

As described above in detail, the abundance of NETs is a fragile balance, which tends to tilt over to a rather deleterious influence during several infectious diseases. To date, several attempts were taken to keep NETs in the right frame. Nevertheless, the combination of DNA with potentially damaging molecules makes it difficult to find the perfect treatment. Here, we summarize the most relevant and promising treatment strategies.

### 5.1. DNase1

Extracellular chromatin and NETs can be digested by naturally occurring DNase1. It dismantles the DNA structure and liberates entangled components, which has to be calculated as a significant risk factor since, e.g., NE or MPO are capable of perpetuating inflammation. However, DNase is the only NET-targeting therapy already in clinical use. It is used for the treatment of virus-associated bronchiolitis [211], as well as cystic fibrosis, in order to improve lung function and reduce the occurrence of infectious exacerbations [212,213]. Similarly, NET DNA in COVID-19 contributes to mucus accumulation, rigidity, and airway occlusion, indicating that severe cases of COVID-19 might also benefit from DNase treatment. A single-center case study was published recently, suggesting that nebulized dornase (recombinant human DNase) reduced supplemental oxygen requirements [214]. Further clinical trials are currently underway investigating the effect of nebulized dornase-α during COVID-19 [215].

### 5.2. Histones

One possible drawback of disentangling DNA fibers could be the subsequent release of histones or proteases, potentially causing cytotoxicity. Recently, a study suggested that the synergy between histone and DNA is critical for sub-lethal signaling [216]. Accordingly, another study proposes a mechanism whereby aggNETs contribute to the detoxification of histones. Neutralization of histones might be a promising target in future, as demonstrated in different murine disease models [217,218]. The C1 esterase inhibitor (C1INH), a serine protease inhibitor, is capable of targeting multiple pathways [219,220] and can bind and neutralize histones due to its glycosylation-dependent overall negative charge. Additional studies revealed that C1INH treatment reduced neutrophil activation and improved inflammation and survival in sepsis patients [221,222]. However, additional preclinical testing and investigation of different disease models is needed to further validate this promising inhibitor as a therapeutic agent during inflammation. Furthermore, a recent study suggests a promising role of an anti-citrullinated protein antibody (tACPA), which prevented NET-associated disease symptoms in different inflammatory pathologies in mice by inhibiting NET formation and increasing NET degradation through macrophages [218]. Accordingly, another study demonstrated that neutralizing citH3 attenuates endothelial damage in vitro and has the capability to improve survival rates and inflammatory responses during LPS-induced sepsis in mice [223].

### 5.3. Neutrophil Elastase

Similar to the abovementioned release of histones, the liberation of NE might also contribute to tissue damage and inflammation since it is capable of disturbing the lung barrier, inducing the release of inflammatory cytokines, thereby fostering a cytokine storm which is often a life-threatening event during ARDS. Small-molecule inhibitors, such as sivelestat [224], alvelestat and Bay-8550, are possible therapeutics directed against NE and currently under investigation. Different studies and clinical trials with ARDS/SIRS patients indicate that sivelestat improves pulmonary function, and shortens the duration of mechanical ventilation and the length of ICU care [225,226], most likely through inhibition of the exaggerated signaling pathways and neutrophil chemotaxis [227,228,229].

### 5.4. Other Treatments

There are several molecules that are able to influence NET formation. Aspirin treatment decreases NET formation in the lung microcirculation and plasma [230] and also decreases the deposition of platelets with neutrophils on the lungs’ vascular walls [231]. TLR-mediated NET formation can be inhibited by the use of blocking antibodies, such as anti-CLEC or the bispecific anti-CLEC5A/TLR2 [232]. Additionally, hydroxychloroquine, also known as an anti-malarial and anti-inflammatory drug, inhibits the stimulation of pDCs by NETs via TLR9 [230]. The antidiabetic drug metformin directly binds the alarmin HMGB1, resulting in increased NET clearance, and attenuates the pro-inflammatory activity of NETs [233,234]. Glucocorticoids, such as dexamethasone, belong to a class of drugs with anti-NET formation activity [235]. Additionally, NET-inhibitory factors have been identified. They specifically inhibit NET formation in vitro and in vivo, thereby suggesting them to be a potential therapeutic agent [236]. Further treatment options exist that do not directly target NET formation but rather neutrophil recruitment. For example, a CXCR2 antagonist reduced neutrophil influx into the airways following an LPS challenge in humans [237]. Nonetheless, blocking neutrophil recruitment always harbors the risk of impairing the innate immune response. In regard to this, a promising therapy might be the use of the CD40 antibody M7, which was shown to limit inflammation without affecting the protective host defense in mice [238]. A summary of possible interventions that are targeted against NETs or their components is listed in Table 1.

## 6. Summary

NETs seem to play a fundamental role in the pathogenesis of several respiratory diseases often attributed to worse outcomes, but the treatment options are scarce. However, in the last years, the knowledge about NET formation increased, and it became certainly clear that there has to be a consensus about the definition of NETs, their stimulation, and their components [239]. Results from experiments with non-physiologic triggers, such as PMA, are most likely not able to represent the in vivo situation. In addition, the detection of NETs should follow certain rules, since only staining of extracellular DNA does not necessarily detect NETs but also other necrotic cell remnants. Inconsistent methods of published studies complicate interpretation of their data. However, much more information about the underlying signaling pathways is required to establish potential therapies. With regard to respiratory diseases, it appears that NETs have a beneficial role in the early phase of disease. They often participate in capturing pathogens and prevent ongoing infection, secondary infections, and dissemination. A number of evasion mechanisms evolved by different bacteria support the importance of this preventive role. Nonetheless, in a later phase, where the disease has manifested, the anti-microbial components of NETs are no longer able to fight infection, but rather contribute to pathology due to their cytotoxic properties: this fragile balance complicates an effective treatment. Studies investigating NETs during different phases of disease are rare. The differentiation of the impact of NETs during onset, progression, and resolution of disease is of great interest and will provide essential contributions to the development of possible therapeutic interventions.

## Figures and Tables

**Table 1:** Summary of NET-targeting therapeutics or compounds in preclinical and clinical applications.

Compound	Target	Application	Reference
Dornase Alfa/DNase	DNA	Bronchiolitis Cystic fibrosis	[211,212,213,214,215]
		Clinical Phase 2 study: COVID19	NCT04359654
C1 esterase inhibitor	Histones	Sepsis patients	[219,220,221,222]
tACPA α-H3-cit	Citrullinated Histones	Inflammatory murine disease models	[218,223]
Sivelestat	Neutrophil elastase	ARDS patients ALI patients	[225,226,227,228,229]
		Clinical phase 4 study: ARDS	NCT00036062
Aspirin αCLEC Glucocorticoids NET-inhibiting factors	Inhibition of NET formation	Critically ill patients Inflammatory murine disease models	[230,231,232,236]
Metformin	HMGB/NET clearance	Diabetes patients	[233,234]
CXCR2 antagonist	Neutrophil recruitment	LPS-challenged humans	[237]
CD40L-M7	Mac1	Inflammatory murine disease models	[238]
